# Differences in Power Acquisition Between Only and Non-only Children: The Effects of Cooperative Orientation, Competitive Orientation, and Dependency on Parents

**DOI:** 10.3389/fpsyg.2022.778726

**Published:** 2022-03-10

**Authors:** Yan Rong, Yulan Han, Linping Dong, Huijuan Bi

**Affiliations:** ^1^College of Business, Shanghai University of Finance and Economics, Shanghai, China; ^2^School of Business, East China University of Science and Technology, Shanghai, China; ^3^Shanghai Bihe Biochemical Technology Co., Ltd., Shanghai, China

**Keywords:** only child, power, cooperative orientation, competitive orientation, dependency on parents

## Abstract

Drawing upon a developmental perspective, we investigated the differences in power acquisition (i.e., rank at work and leader role occupancy in university) between only and non-only children as well as the mediating role of cooperative and competitive orientations and the moderating role of dependency on parents. To test our hypotheses, we conducted two field studies in 155 part-time Master of Business Administration (MBA) students (Study 1) and 375 senior students (Study 2). Results showed that: (1) non-only children were more likely to achieve higher rank at work than only children; (2) only children were less likely than non-only children to acquire power in organizations because they scored lower in cooperative orientation; however, the mediating effect of competitive orientation was not significant; (3) the difference in cooperative orientation between only and non-only children was smaller when dependency on parents was high, whereas it became larger when dependency on parents was low. Our research contributes to the understanding of how family structure influences individual power acquisition.

## Introduction

Numerous researchers have investigated the antecedents of power acquisition, such as physical characteristics, personalities, needs, and motivations (Keltner et al., 2003; Galinsky et al., 2015). However, few have considered the effect of early family experiences in predicting the capability of gaining power. Indeed, power acquisition is a dynamic process across the lifespan, and the factors that determine an individual’s power may derive from early family life (Andeweg and Berg, 2003). It has been shown that family factors inevitably influence the development of sociability and leadership (Trent and Spitze, 2011; Liu et al., 2019), which are associated with elevated power (Keltner et al., 2003; Reitan and Stenberg, 2019).

One of the most profound early-life experiences is growing up with siblings (Falbo and Polit, 1986; Feng, 2000; Cameron et al., 2013); however, a large number of only children are deprived of such an experience. It is estimated that there were over 220 million only children in mainland China at the end of 2015 (Li et al., 2018); moreover, the number of American women who decided to have only one child doubled from 11% in 1976 to 22% in 2015 (Gibson, 2019). How the unique family experience of being an only child shapes their power acquisition remains unclear. Researchers have found that only children are overrepresented among incumbents in political office (Andeweg and Berg, 2003; Reitan and Stenberg, 2019), whereas others have revealed that only children adapt poorly to social activities (Kitzmann et al., 2002) or have lower self-rated sociability (Falbo and Polit, 1986). Given that the number of single-child families is continuing to increase (Feng, 2020), explorations on the influence of only child status on power acquisition are needed.

We propose that only children may be disadvantaged due to being deprived of sibling interactions (Xiao and Feng, 2010), which would have provided a ‘training ground’ for power struggles later in life (Jiao et al., 1986; Newman and Taylor, 1994; Cameron et al., 2013). The lack of experience in competing and coordinating with siblings may lower their competitive and cooperative orientations (Mander, 1991; Roberts and Blanton, 2001; Sulloway, 2001), which are important factors to enable domination and acquisition of power (Keltner et al., 2008; Galinsky et al., 2015). As suggested by socioanalytic theory, getting along (cooperation) and getting ahead (competition) are two promising and compatible paths for leader emergence and power acquisition (Hogan and Holland, 2003; Marinova et al., 2013). Therefore, we predicted that cooperative and competitive orientations are important mechanisms that explain the differences in power acquisition between only and non-only children.

In addition, from a developmental perspective, child-parental relationships significantly affect the growth and socialization of children (Liu et al., 2010, 2020). We propose that dependency on parents interacts with only child status to influence behavioral orientations. If children are highly dependent on their parents, non-only children may have less autonomy to solve problems through sibling interactions; however, they may compete more with their siblings for resources and attention from parents. Therefore, we hypothesize that as dependency on parents increases, the difference in cooperative orientation between non-only and only children will be smaller, whereas the difference in competitive orientation will be larger.

**FIGURE 1:**
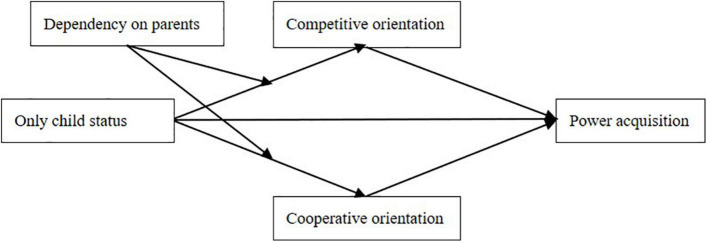
Theoretical model.

To summarize, this paper tries to explore the difference in power acquisition between only and non-only children and examine the mediating role of cooperative and competitive orientations as well as the moderating role of dependency on parents (Figure 1). We aim to contribute to the field in several ways. First, power acquisition reflects individuals’ social adaptabilities at different life stages (i.e., school years and career stage). By combining the studies on the family backgrounds with power literature from a developmental perspective, our paper provides a deeper understanding of the antecedents of power acquisition. Secondly, this research explores the mediating effects of cooperative and competitive orientations and offers possible explanations for the difference in power acquisition between only and non-only children. Although researchers have explored the impact of being an only child on several social activities (Falbo, 2012; Cameron et al., 2013), they paid less attention to the underlying mechanisms. This paper provides a novel explanation for the relationship between only child status and social adaptabilities. Finally, identifying the moderating effect of dependency on parents can help us to understand whether sibling and child-parental relationships interactively influence individual behaviors. Child-parental relations and sibling relations were identified as two key mechanisms to explain the behavioral pattern of the only child (Falbo and Polit, 1986; Reitan and Stenberg, 2019), yet their interactive relationships were rarely considered. We demonstrate that siblings and parents are two sources of socialization and the effect of sibling deprivation (being an only child) is conditioned upon the child-parental relations.

## Theoretical Background and Research Hypotheses

### Only Child Status and Power Acquisition

Power refers to the asymmetrical control of valuable resources in social relationships (Magee and Galinsky, 2008; Rucker and Galinsky, 2017). Power acquisition is reflected by an individual’s position in an organizational hierarchy (i.e., rank) and role in the group (i.e., leader role) among others (Galinsky et al., 2015). From a developmental perspective, compared with non-only children, only children lack the experience of interacting with siblings, which may disadvantage them in teamwork situations and the capability of gaining power in group settings.

Sibling interactions provide children with teamwork experience with peers, which helps develop their abilities to work effectively in a team. Non-only children need to resolve conflicts with siblings, distribute resources among siblings, and coordinate with each other to complete group tasks (Hughes et al., 2018). Such experiences aid in their development of communication, coordination, and conflict resolution skills (Mancillas, 2006). Only children who lack these learning experiences in the family environment may be at a disadvantage. For example, studies have found that only children are more aggressive and less popular among classmates and have poorer performance in conflict resolutions (Kitzmann et al., 2002) and poorer interpersonal skills (Downey and Condron, 2008). Furthermore, Cameron et al. (2013) highlighted that parents of non-only children educate their children to share resources with and trust their siblings, whereas these educational experiences are less common in families with only one child.

These differences in growth experiences likely influence an individual’s power acquisition within a group setting. According to the reciprocal influence model of social power (Keltner et al., 2008), power is usually granted to individuals who can push the team to achieve its goals and contribute to the team. Indeed, individuals who are competent at resolving team conflicts, setting good examples, and distributing resources fairly are more likely to obtain power within the team (Aureli and de Waal, 2000). Therefore, non-only children may be more effective working in a team and eventually occupying leadership roles than only children. In addition, non-only children have more opportunities to develop interpersonal influence tactics than only children. Within families, parents usually have high power and control valuable resources, whereas children typically have low power and accept resource allocation (Recchia et al., 2010). Because only children primarily interact with high-power parents, they have fewer opportunities to participate in family decision-making. In contrast, non-only children have numerous opportunities to make decisions together with their siblings who have similar levels of power. Such experiences benefit the development of leadership skills and influence tactics, which will aid in acquiring power in the future (i.e., at school and various career stages). Thus, we proposed:

*Hypothesis 1. Compared with non-only children, only children are less likely to gain power within group settings.*

### The Mediating Roles of Cooperative and Competitive Orientation

We further propose that, cooperative and competitive orientation will explain the disadvantages of only children in power acquisitions. According to Chen et al. (2011), cooperative and competitive orientations are “stable individual differences regarding people’s beliefs about and attitudes toward the nature of their relationship with others”(p.354). Specifically, cooperative orientation refers to individuals’ view of others as interdependent partners and their willingness to work with others. Competitive orientation refers to individuals’ view of others as means for self-development and to demonstrate self-worth.

Chen et al. (2011) suggested that among Chinese people, cooperative and competitive orientations are two distinct yet not opposing concepts. Traditional Western research often defines cooperation and competition as two ends of one continuum (Deutsch, 1949a,b). However, people living in groups are often involved in mixed-motive situations, whereby a conflict exists within an individual between maximizing individual interest and maximizing collective interest (Chen et al., 2011; Gnyawali and Ryan Charleton, 2018). Consequently, it is possible for individuals to simultaneously cooperate and compete with others. Chen et al. (2011) proposed that Chinese people, who are capable of dialectical thinking, tend to perceive cooperation and competition as coexisting rather than opposing. They found that cooperative and competitive orientations are distinct traits that differentially affect peoples’ cognition and behavior (Chen et al., 2011). Therefore, we treated cooperative and competitive orientations as coexisting rather than mutually exclusive concepts.

According to the socioanalytic theory, getting along (cooperation) and getting ahead (competition) are two fundamental motives within social groups and two promising mechanisms for leader emergence (Hogan and Holland, 2003; Marinova et al., 2013). This is because the pervasive use of work teams in modern organizations requires individuals to simultaneously cooperate and compete with their team members. Due to the interdependent nature of teamwork, members must cooperate with one another to effectively complete tasks and achieve common goals; however, at the individual level, the need to constantly outperform others and improve one’s performance remains strong. Thus, both getting along (cooperation) and getting ahead (competition) contribute to leader emergence and power affordance (Marinova et al., 2013). Integrating these views, we offer both cooperative orientation and competitive orientation as unique mechanisms linking only child status to power acquisition.

First, we propose that only children are less likely to gain power in the group as they have lower cooperative orientation than non-only children. Individuals’ socialization process shapes their cooperative orientation (Knight et al., 1993), and the family growth experience is an important component of socialization (Parke and Buriel, 1998). The experience of mutual assistance, resource sharing, and cooperation between siblings affects an individual’s tendency to cooperate (Van Lange et al., 1997); only children lack this socialization process within the family environment. For example, when playing, siblings negotiate on sharing toys, dividing tasks, and working together to accomplish play goals (Hughes et al., 2018). Therefore, sibling interactions provide an effective training ground for learning to share and collaborate.

Previous studies have found that only children prefer to be alone over participating in team activities (Claudy, 1979), and they rate themselves as less social than non-only children (Falbo and Polit, 1986). In addition, because only children do not need to share resources or cooperate and solve problems with siblings, they lack experience in perspective-taking and are likely to be ego-centric. For instance, when only children are asked to recall their growth experiences, they recall more self-focused memories and fewer collective descriptions (Wang et al., 1998). This is not conducive to interpersonal cooperation during adulthood (Roberts and Blanton, 2001). In contrast, individuals who grow up in a multiple-child family are more likely to believe that people are interdependent (Bender and Chasiotis, 2011) and exhibit more cooperative behaviors in group settings (Madsen and Shapira, 1977; Van Lange et al., 1997), such as helping others, participating in teamwork, and taking charge (Jiao et al., 1986).

Individuals with a high cooperative orientation are willing to communicate with others, which helps them form harmonious interpersonal relationships and gain influence within the group. Furthermore, they are more willing to contribute to team goals and exhibit more organizational citizenship behaviors (Chen et al., 2011), which are beneficial for being recognized by and acquiring power within the group (Anderson et al., 2001; Keltner et al., 2008; Willer, 2009) or being elected as a team leader (Marinova et al., 2013). In practice, cooperative orientation is an important criterion in selection and promotion decisions, and individuals with high cooperativeness are more likely to be promoted than those with low cooperativeness (Judge et al., 2002; Hogan and Holland, 2003). Therefore, we predict that individuals with a high cooperative orientation are more likely to gain power within the group than are those with a low cooperative orientation.

In summary, compared with non-only children, only children lack the experience of interacting with siblings, which may lead to a lower cooperative orientation and in turn, hinder their performance in social groups and weaken their capability to gain power. Therefore, we propose:

*Hypothesis 2. The effect of only child status on power acquisition is mediated by cooperative orientation.*

Second, we propose that, only children are less likely to gain power in the group as they have lower competitive orientation than non-only children. Individuals’ competitive orientation is shaped by their growth environment and family life experiences (Van Lange et al., 1997; Booth et al., 2019). From a Darwinian perspective, children are generally in conflict with their siblings over the allocation of parental investment to satisfy the needs of survival and development (Sulloway, 2010). In families with multiple children, parents need to allocate limited energy and resources among their children, and children compete for attention, care, and material input from their parents (Rebar et al., 2020; Wang and Feng, 2021). Children are always sensitive to parental love, affection, and support (Eddleston and Kidwell, 2012).

Sibling rivalry in the family affects an individual’s competitive orientation (Sulloway, 2001). As family size increases, the time and energy that parents invest in each child gradually disperse, and competition over resources becomes fiercer (Lawson and Mace, 2009). Children with siblings realize that in order to obtain sufficient resources, they must beat the competition (Bengtsson et al., 2016). This experience likely underpins their competitive orientation. In contrast, only children obtain parental attention and resources without needing to compete (Sulloway, 2001). Therefore, their inclination to compete is lower (Locke and Latham, 1990). Indeed, Cameron et al. (2013) found that compared with non-only children, only children are less inclined to participate in competitive games.

Individuals with a high competitive orientation tend to maximize outcomes over others (Van Lange, 1999) to obtain rewards, status, or power. For example, individuals’ competitive personality is positively correlated with their need for power (Lu et al., 2013). Children who tend to compete with their siblings are more likely to gain leadership positions in their relationships with friends (Stocker and Dunn, 1990). Moreover, employees with a high competitive orientation are more likely to become team leaders (Marinova et al., 2013).

In summary, compared with non-only children, only children lack the experience of competing with siblings in the family, which may lead to lower competitive orientation and in turn, reduce their possibility of gaining power. Therefore, we propose:

*Hypothesis 3. The effect of only-child status on power acquisition is mediated by competitive orientation.*

### The Moderating Effect of Dependency on Parents

From a developmental perspective, child-parental interactions are a key factor that affects individual socialization (Parke and Buriel, 1998; Liu and Jiang, 2021). Moreover, sibling interactions and child-parental interactions may interactively influence children’s socialization (Mounts, 2002). Because children’s dependency on their parents is an important reflection of child-parental interactions, the current research examines whether dependency on parents moderates the effect of only child status on cooperative/competitive orientation.

Children who are highly dependent on their parents rely on parents for advice, help, and resources; thus, their orientation to cooperate with siblings to seek solutions may be lower. Studies have found that parental interference during conflict resolutions among children deprives children of the opportunity to solve problems on their own (Bank and Kahn, 1997; Avloniti et al., 2014). Furthermore, the overparenting of adolescents can damage their social adaptation and interpersonal interactions (Liu et al., 2019). These findings suggest that children who are overly dependent on their parents, even those with siblings, are less likely to develop interpersonal and cooperative skills through communicating and negotiating in the family (Ross et al., 1996). Therefore, the difference in cooperative orientation between only and non-only children will be smaller as dependency on parents increases.

In contrast, when dependency on parents is low, non-only children have more opportunities to resolve conflicts with siblings, consider others’ perspectives and interests, and collaborate with siblings to achieve goals (Ross et al., 1996). Under such conditions, non-only children are more likely to develop higher cooperative orientation; thus, differences in cooperative orientation between only and non-only children will be larger. Research on animal families lends support to this prediction. For instance, when no care is provided by parents, larvae evolve to be more cooperative (Rebar et al., 2020). Therefore, we propose:

*Hypothesis 4a. Dependency on parents moderates the effect of only child status on cooperative orientation: when dependency on parents is high, the difference in cooperative orientation between only and non-only children will be smaller; when dependency on parents is low, the difference will be larger.*

Drawing on Hypotheses 3 and 4a, we further proposed the following:

*Hypothesis 4b: The indirect effect of only-child status on power acquisition through cooperative orientation will be stronger when dependency on parents is low (vs. high).*

We further predicted that dependency on parents moderates the effect of only child status on competitive orientation. When children are highly dependent on parents, the competition over resources and attention from parents becomes fiercer for non-only children (Hudson and Trillmich, 2008), which will further strengthen their competitive orientation. Gibson and Lawson (2011) believe that children’s dependency on parental resources intensifies their competition for resources within the family. Studies on family business also revealed that children who are dependent on their parents for conflict resolution during childhood experience difficulties in resolving conflicts on their own during adulthood, which in turn exacerbates sibling rivalries (Friedman, 1991; Avloniti et al., 2014). Similarly, research on animal families found that if parents provide their offspring with complete care, they evolve to be more competitive over resources that parents supply than those with no care (Rebar et al., 2020).

However, when dependency on parents is low, children are less motivated to compete among siblings for parental resources and attention. In line with this, evolutionary biology studies have found an interactive relationship between brood size and parental food provision; when offspring are dependent on parental food, competition intensifies with the increase in brood size, whereas when offspring are independent, the relationship weakens (Smiseth et al., 2007). Therefore, we predicted that when dependency on parents is high, the difference in competitive orientation between non-only and only children will be larger, whereas when dependency on parents is low, the difference will be smaller. Therefore, we proposed:

*Hypothesis 5a: Dependency on parents moderates the effect of only child status on competitive orientation: when dependency on parents is high, the difference in competitive orientation between non-only and only children will be larger; when dependency on parents is low, the difference will be smaller.*

Combining Hypotheses 3 and 5a, we further proposed the following:

*Hypothesis 5b: The indirect effect of only child status on power acquisition through competitive orientation will be stronger when dependency on parents is high (vs. low).*

### Research Overview

To test our hypotheses, we conducted two field studies by collecting data from 155 part-time MBA students (Study 1) and 375 senior students (Study 2). In Study 1, rank within the organization was used to capture power acquisition in the workplace. In Study 2, leader role occupancy in the university was used to capture power acquisition during one’s schooling years.

## Study 1

### Methods

#### Sample and Procedure

In Study 1, we collected responses from 155 part-time MBA students from a University in eastern China using a multi-wave research design^1^. At Time 1, we distributed the survey to 606 MBA students and received 563 responses. Participants provided background information, which included age, sex, tenure, industry, firm size, and only child status. Six months later (Time 2), we asked participants to report their cooperative and competitive orientations and received 460 responses. Eighteen months later (Time 3), participants reported their rank at work, which reflected their power acquisition within the workplace, for which we received 164 responses. We matched the responses from the three waves and received a final total of 155 responses. The response rate was 25.58%.

Of the 155 participants, 47.74% were male. Average age was 32.32 years (*SD* = 4.10), average employment years was 8.19 years (*SD* = 3.83), and 61.94% of them were only children. The final sample was not significantly different from the subjects with incomplete data in terms of sex [*F*(1, 561) = 0.05, *p* = 0.83], age [*t*(561) = –0.41, *p* = 0.69], only child status [*F*(1, 458) = 0.39, *p* = 0.53], and rank [*t*(500) = –1.11, *p* = 0.27].

#### Measures

We used a five-point Likert scale for the survey (1 = *strongly disagree*; 5 = *strongly agree*). For only child status, participants reported whether or not they are an only child (0 = non-only child; 1 = only child). For cooperative and competitive orientations, we used a 13-item scale developed by Chen et al. (2011). A sample item for cooperative orientation (7 items) was “It is important to coordinate with others at work,” and Cronbach’s alpha was 0.77. A sample item for competitive orientation (6 items) was “I feel somewhat disappointed when others perform better than me,” and Cronbach’s alpha was 0.70. To assess rank, participants were asked to report their rank in their organization out of one of four levels: 1 = non-management, 2 = line management, 3 = middle management, 4 = senior/executive management, which is a common method to measure structural power (Anderson et al., 2008). Sex, age, employment years, firm size, and industry were included as control variables, as they are found to be influential factors for power acquisition (Kent and Moss, 1994; Truninger et al., 2021). Because 56.13% of the participants came from the finance industry, we coded this industry as a dummy variable (0 = others; 1 = finance).

### Results

#### Descriptive Analysis

Table 1 shows the descriptive statistics and correlations among the variables. Only child status was negatively correlated with rank (*r* = –0.28, *p* < 0.001), and cooperative orientation (*r* = –0.26, *p* = 0.01) but was not significantly correlated with competitive orientation (*r* = 0.06, *p* = 0.47). Cooperative orientation was positively correlated with rank (*r* = 0.31, *p* < 0.001), whereas the correlation between competitive orientation and rank was not significant (*r* = 0.00, *p* = 0.97).

**TABLE 1:** Means, standard deviations, and correlations of the research variables (Study 1).

Variables	*M*	*SD*	1	2	3	4	5	6	7	8
1 Sex	0.48	0.50	—							
2 Age	32.32	4.10	0.12	—						
3 Employment years	8.19	3.83	0.16*	0.75**	–0.10	—				
4 Lg (firm size)	2.70	1.07	0.06	–0.11	−0.26**	—				
5 Finance industry	0.56	0.50	0.12	−0.37***	−0.16^†^	0.21**	—			
6 Only child status	0.62	0.49	–0.02	−0.16^†^	0.21**	0.23**	0.19*	—		
7 Cooperative orientation	4.01	0.55	–0.06	0.30***	0.02	–0.04	−0.25**	−0.26**	(0.77)	
8 Competitive orientation	3.38	0.65	0.05	–0.06	0.40**	0.09	–0.05	0.06	–0.07	(0.70)
9 Rank	2.25	0.93	0.06	0.44***	–0.10	−0.28***	−0.32***	−0.28***	0.31***	0.00

N = 155; ^†^p < 0.10, *p < 0.05, **p < 0.01, ***p < 0.001.

#### Hypothesis Testing

As shown in Table 2 (Model 2), only child status had a significant negative effect on rank (*B* = –0.28, *t* = –2.04, *p* = 0.04), which supported Hypothesis 1.

**TABLE 2:** Results of the regression analysis (Study 1).

Variable	Rank			Cooperative orientation	Competitive orientation
			
	Model 1	Model 2	Model 3	Model 4	Model 5
Sex	0.07 (0.13)	0.06 (0.13)	0.08 (0.13)	−0.09 (0.09)	0.08 (0.11)
Age	0.06 (0.02)*	0.06 (0.02)*	0.05 (0.02)*	0.04 (0.02)*	−0.03 (0.02)
Employment years	0.03 (0.03)	0.03 (0.03)	0.03 (0.03)	−0.00 (0.02)	0.03 (0.02)
Lg (firm size)	−0.18 (0.06)**	−0.16 (0.06)*	−0.17 (0.06)**	0.03 (0.04)	0.06 (0.05)
Finance industry	−0.28 (0.14)^†^	−0.25 (0.14)^†^	−0.20 (0.14)	−0.14 (0.09)	−0.16 (0.12)
Only child status		−0.28 (0.14)*	−0.22 (0.14)	−0.24 (0.09)**	0.07 (0.11)
Cooperative orientation			0.27 (0.13)*		
Competitive orientation			0.06 (0.10)		
*F*	11.60***	10.57***	8.68***	4.80***	0.98
*R* ^2^	0.28	0.30	0.32	0.16	0.03

N = 155; ^†^p < 0.10, *p < 0.05, **p < 0.01, ***p < 0.001.

As shown in Models 4 and 5, only child status had a significant negative effect on cooperative orientation (*B* = –0.24, *t* = –2.75, *p* = 0.007) but not competitive orientation (*B* = 0.07, *t* = 0.64, *p* = 0.53). The parallel mediators model was used to test the mediating effects of cooperative and competitive orientation (Preacher and Hayes, 2004). According to Model 3, cooperative orientation had a significant positive effect on rank (*B* = 0.27, *t* = 2.14, *p* = 0.03), but the effect of competitive orientation on rank was not significant (*B* = 0.06, *t* = 0.60, *p* = 0.55). The indirect effect of only child status on rank through cooperative orientation was negative and significant (a*b = –0.06, 95%CI = [–0.18, –0.01]), whereas that through competitive orientation was not significant (a*b = 0.00, 95% CI = [–0.01, 0.05]). Therefore, Hypothesis 2 was supported but Hypothesis 3 was not supported.

#### *Post hoc* Analysis

We created two subgroups for all only children and non-only children. In each of the group, we performed independent *t*-tests to compare their cooperative/competitive orientation between those who have high power and those who have low power within two subgroups.

Specifically, those who were in a middle or top manager position were classified into the high-power group, and all remaining participants were classified into the low-power group. We found that in the only children group, those with high power (*n* = 27) reported higher cooperative orientation than those who have low power [*n* = 69; *t*(94) = 2.51, *p* = 0.01]. Similarly, in the group of non-only children, those who have high power (*n* = 34) reported higher cooperative orientation than those who have low power [*n* = 25; *t*(57) = 2.51, *p* = 0.01]. However, the difference in competitive orientation was not significant for only children [*t*(94) = –0.95, *p* = 0.35] or non-only children [*t*(57) = 0.47, *p* = 0.64].

### Discussion

Study 1 provided direct support for a main effect of only child status on power acquisition, as well as the mediating effect of cooperative orientation. We showed that, only children are less likely to acquire a high level of power than non-only children because they have lower cooperative orientation. However, because MBA students are working adults and thus their dependency on parents are limited, this sample is not appropriate to test the moderating effect of dependency on parents. Besides, cooperative and competitive orientation reflect people’s individual attributes that influence power acquisition, while the process of becoming powerful is also influenced by the motivation. Therefore, we conducted another field study in which we tested the moderating role of dependency on parents and tried to rule out the motivational path by controlling for need for dominance in our statistical model.

## Study 2

### Methods

#### Sample and Procedure

In Study 2, we collected data from senior students. Participants reported their only child status, cooperative orientation, competitive orientation, dependency on parents, and student leadership status at university. A leader role within student associations reflects power acquisition during schooling years (Guinote, 2017). We distributed surveys to 564 senior students and received 388 responses. After excluding those with incomplete data, we obtained 375 valid responses (valid response rate = 66.49%). Of these participants, 35.73% were male, the average age was 21.31 years (*SD* = 0.73), and 65.60% were only children.

#### Measures

A five-point Likert scale was used for the survey (1 = *strongly disagree*; 5 = *strongly agree*). Only child status, cooperative orientation (α = 0.86), and competitive orientation (α = 0.81) were captured using the same measures as those used in Study 1. To measure dependency on parents, we used a four-item scale developed by Steinberg and Silverberg (1986). A sample item was “I go to my parents for help before trying to solve a problem myself.” Cronbach’s alpha was 0.84. For leader role occupancy, students who assumed leadership positions, such as classroom monitor and student union president, were given a score of 1, whereas those who did not were given a 0.

Sex, age, department (accounting, economics and finance, business administration, or management science), *hukou*^2^ (0 = urban; 1 = rural), and family income per month were included as control variables. In addition, because need for dominance is an important motive that influences power acquisition (Fagenson, 1992), this was controlled for in Study 2. A five-item scale adopted from Steers and Braunstein (1976) was used to measure need for dominance. A sample item was “I strive to gain more control over the events around me at work,” and Cronbach’s alpha was 0.91.

### Results

#### Descriptive Analysis

Table 3 shows the descriptive statistics and correlations among the variables. Only child status was negatively correlated with cooperative orientation (*r* = –0.13, *p* = 0.01), but was not significantly correlated with competitive orientation (*r* = 0.04, *p* = 0.46), leader role occupancy (*r* = –0.03, *p* = 0.58), or need for dominance (*r* = –0.01, *p* = 0.81). Cooperative orientation was positively correlated with leader role occupancy (*r* = 0.19, *p* < 0.001), whereas the correlation between competitive orientation and leader role occupancy was not significant (*r* = 0.03, *p* = 0.56). Besides, need for dominance is positively related to leader role occupancy (*r* = 0.19, *p* < 0.001).

**TABLE 3:** Means, standard deviations, and correlations of the research variables (Study 2).

Variables	*M*	*SD*	1	2	3	4	5	6	7	8	9
1 Sex	0.36	0.48	—								
2 Age	21.31	0.73	0.21***	—							
3 Hukou	0.31	0.46	0.03	0.08	—						
4 Family income	2.40	0.98	0.02	–0.01	−0.36***	—					
5 Need for dominance	2.90	0.67	–0.02	–0.01	–0.05	0.13*	(0.91)				
6 Only child status	0.66	0.48	0.04	–0.03	−0.42***	0.17**	–0.01	—			
7 Cooperative orientation	3.47	0.60	−0.14**	0.01	0.04	0.03	0.27***	−0.13*	(0.86)		
8 Competitive orientation	3.03	0.56	0.03	–0.03	0.03	0.01	0.32***	0.04	0.23***	(0.81)	
9 Dependency on parents	2.68	0.69	0.09^†^	−0.11*	–0.02	0.06	0.29***	0.05	–0.04	0.30***	(0.84)
10 Leader role occupancy	0.53	0.50	–0.07	–0.03	–0.08	0.03	0.19***	–0.03	0.19***	0.03	−0.08

N = 375; ^†^p < 0.10, *p < 0.05, **p < 0.01, ***p < 0.001.

#### Hypothesis Testing

As shown in Table 4 (Model 7), the effect of only child status on leader role occupancy was not significant (*B* = –0.25, *z* = –1.00, *p* = 0.32); thus, Hypothesis 1 was not supported.

**TABLE 4:** Results of the regression analysis (Study 2).

Variable	Leader role occupancy				Cooperative orientation		Competitive orientation	
			
	Model 6	Model 7	Model 8	Model 9	Model 10	Model 11	Model 12	Model 13
Sex	−0.23 (0.23)	−0.22 (0.23)	−0.12 (0.24)	−0.08 (0.24)	−0.18 (0.07)**	−0.16 (0.06)*	0.07 (0.06)	0.03 (0.06)
Age	−0.08 (0.15)	−0.08 (0.15)	−0.11 (0.15)	−0.16 (0.16)	0.03 (0.04)	0.02 (0.04)	−0.04 (0.04)	−0.02 (0.04)
Accounting	−0.03 (0.32)	−0.01 (0.32)	0.04 (0.32)	0.04 (0.32)	−0.03 (0.09)	−0.02 (0.09)	0.12 (0.08)	0.11 (0.08)
Economics and Finance	−0.48 (0.31)	−0.45 (0.31)	−0.40 (0.31)	−0.35 (0.31)	−0.08 (0.09)	−0.07 (0.08)	0.08 (0.08)	0.05 (0.08)
Business administration	0.21 (0.31)	0.22 (0.31)	0.28 (0.32)	0.34 (0.32)	−0.04 (0.09)	−0.02 (0.09)	0.13 (0.08) ^†^	0.11 (0.08)
Hukou	−0.42 (0.25) ^†^	−0.53 (0.27) ^†^	−0.52 (0.28) ^†^	−0.51 (0.28) ^†^	−0.00 (0.08)	−0.00 (0.07)	0.08 (0.07)	0.07 (0.07)
Family income	−0.08 (0.12)	−0.08 (0.12)	−0.09 (0.12)	−0.08 (0.12)	0.01 (0.03)	0.01 (0.03)	−0.01 (0.03)	−0.01 (0.03)
Need for dominance	0.62 (0.17)***	0.62 (0.17)***	0.57 (0.18)**	0.68 (0.19)***	0.24 (0.04)***	0.26 (0.05)***	0.27 (0.04)***	0.22 (0.04)***
Only child status		−0.25 (0.25)	−0.16 (0.26)	−0.15 (0.26)	−0.15 (0.07)*	−0.14 (0.07)*	0.08 (0.06)	0.07 (0.06)
Cooperative orientation			0.56 (0.20)**	0.46 (0.21)*				
Competitive orientation			−0.20 (0.22)	−0.10 (0.22)				
Dependency on parents				−0.65 (0.30)*		−0.30 (0.07)***		0.15 (0.07)*
Only child status × Dependency on parents				0.41 (0.36)		0.32 (0.09)***		0.04 (0.08)
–2Log likelihood	−247.58**	−247.07**	−242.92***	−240.03***				
*R* ^2^					0.11***	0.15***	0.12***	0.16***
Δ*R*^2^						0.04***		0.04***

N = 375; ^†^p < 0.10, *p < 0.05, **p < 0.01, ***p < 0.001.

The parallel mediators model was used to test the mediating effects of cooperative orientation and competitive orientation (Preacher and Hayes, 2004). As shown in Models 10 and 12, only child status had a significant negative effect on cooperative orientation (*B* = –0.15, *t* = –2.18, *p* = 0.03) but not competitive orientation (*B* = 0.08, *t* = 1.26, *p* = 0.21). According to Model 8, cooperative orientation had a significant positive effect on leader role occupancy (*B* = 0.56, *z* = 2.77, *p* = 0.01), but the effect of competitive orientation on leader role occupancy was not significant (*B* = –0.20, *z* = –0.93, *p* = 0.35). The indirect effect of only child status on leader role occupancy through cooperative orientation was negative and significant (a*b = –0.09, 95% CI = [–0.24, –0.01]), whereas that through competitive orientation was not significant (a*b = –0.02, 95% CI = [–0.11, 0.01]). Thus, Hypothesis 2 was supported and Hypothesis 3 was not supported.

**FIGURE 2:**
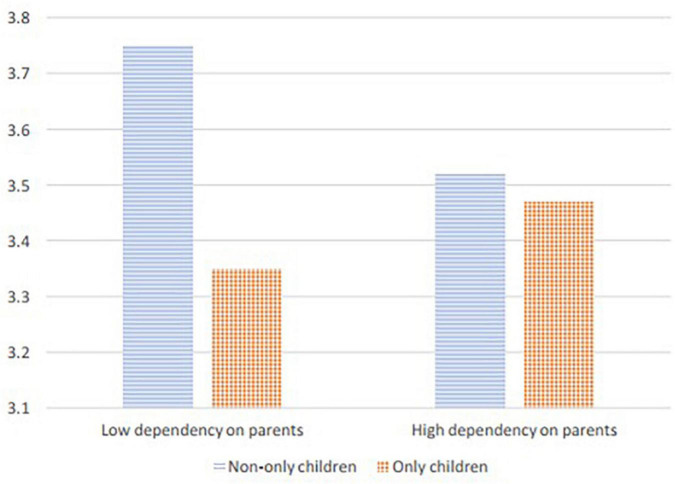
Moderating effect of only child status.

We revealed a significant interaction effect of only child status and dependency on parents on cooperative orientation (Table 4, Model 11; *B* = 0.32, *t* = 3.61, *p* < 0.001) but not on competitive orientation (Table 4, Model 13; *B* = 0.04, *t* = 0.48, *p* = 0.63). As shown in Figure 2, the negative effect of only child status on cooperative orientation became non-significant when dependency on parents was high (*B* = 0.08, *t* = 0.86, *p* = 0.39) but became stronger when dependency on parents was low (*B* = –0.36, *t* = –3.99, *p* < 0.001). Thus, Hypothesis 4a was supported, whereas Hypotheses 5a and 5b were not supported.

We used Preacher et al.’s (2007) method to calculate the point estimate and bias-corrected confidence interval for the moderated mediation effect. The bootstrap results showed that the indirect effect of only child status on leader role occupancy mediated by cooperative orientation was negative and significant (a*b = –0.21, 95% CI = [–0.44, –0.05]) when dependency on parents was low; however, this indirect was not significant (a*b = 0.05, 95% CI = [–0.05, 0.19]) when dependency on parents was high. Hypothesis 4b was thus supported.

#### *Post hoc* Analysis

Similar to Study 1, we also created two subgroups for all the only children and non-only children. In each of the group, we performed independent *t*-tests to compare their cooperative/competitive orientation between those who have high power and those who have low power within two subgroups.

Specifically, those who occupy a leader role in university were classified into the high-power group, and all remaining were classified into the low-power group. We found that in the only children group, those who have high power (*n* = 128) reported higher cooperative orientation than those who have low power [*n* = 118; *t*(244) = 2.36, *p* = 0.02]. Similarly, in the group of non-only children, those who have high power (*n* = 71) reported higher cooperative orientation than those who have low power [*n* = 58; *t*(127) = 3.19, *p* = 0.002]. However, the difference in competitive orientation was not significant for only children [*t*(244) = 0.57, *p* = 0.49] or non-only children [*t*(127) = 0.03, *p* = 0.98].

#### Discussion

Study 2 offered strong support for our predictions that only children have disadvantages in power acquisition because they have lower cooperative orientation. Furthermore, it showed a moderating effect of dependency on parents on the relationship between only child status and cooperative orientation. Besides, by controlling for need for dominance, we could rule out the alternative explanation that the difference in power acquisition between only and non-only children is due to the difference in needs for power.

## General Discussion and Conclusion

By collecting data from 155 part-time MBA students (Study 1) and 375 senior students (Study 2), we explored the difference in power acquisition between only and non-only children as well as the mechanisms and boundary conditions. Results showed that non-only children were more likely to acquire power than were only children. This is because only children were deprived of sibling interactions in the family environment, which resulted in a lower cooperative orientation. Furthermore, the difference in cooperative orientation between only and non-only children became smaller when dependency on parents was high, whereas the difference became larger when dependency on parents was low.

### Theoretical Contributions

This study contributes to the literature in three ways. First, from a developmental perspective, this research links power literature with research on family structure and deepens our understanding of the differences in power acquisition between only and non-only children. Researchers have investigated the social adaptability of only children extensively; however, these studies focused primarily on their social participation, emotional intelligence, and conflict resolution (Falbo, 2012; Downey et al., 2015) and neglected their power acquisition outcomes. Power reflects individuals’ position in the social hierarchy and their control of valuable resources (Anderson and Brion, 2014), which is an important outcome of social adaptation. Investigating the difference in power acquisition between only and non-only children expands our understanding of the social adaptation of only children. Furthermore, research on power acquisition has rarely focused on individuals’ family growth experiences during childhood. By adopting a developmental perspective (Liu et al., 2021), we revealed a difference in power acquisition between only and non-only children based on their early growth experiences, which demonstrated that the capability of acquiring power develops throughout an individual’s lifespan.

Second, this research uncovered the mechanism underlying the difference in power acquisition between only and non-only children: the mediating effect of cooperative orientation. Our results support the notion that sibling interactions provide critical learning experiences for non-only children (Falbo and Polit, 1986; Wang et al., 1998). Moreover, the absence of siblings results in a lack of cooperation experience in the family environment, which weakens only children’s development of cooperative orientation and in turn, decreases their capability to acquire power. In contrast, the mediating effect of competitive orientation was not significant, which may be because Chinese culture emphasizes collectivism and harmonious relationships, which favors prosocial cooperative behaviors over competitive behaviors (Cox et al., 1991; Marcus and Le, 2013; Gneezy et al., 2016).^3^ Therefore, individuals who exhibit more cooperative behaviors are more likely to be afforded power than those exhibiting more competitive behaviors (Ramamoorthy and Flood, 2002; Brewer and Chen, 2007; Leibbrandt et al., 2013). Consequently, cooperative orientation can better explain the difference in power acquisition between only and non-only children in the Chinese context.

Finally, this study broadens our understanding of the development of cooperative orientation by combining parental and sibling perspectives (Andeweg and Berg, 2003). Previous studies have predominantly focused on how individual characteristics, such as sex and age, influence the difference between only and non-only children (Trent and Spitze, 2011; Liu and Jiang, 2021). In this research, we found that dependency on parents and only child status interactively influenced individual cooperative orientation, which suggests that child-parental interactions moderate the effects of sibling interactions (Falbo and Polit, 1986; Cameron et al., 2013). Thus, considering both parental influence and sibling interactions may help us to better understand the differences between only and non-only children.

### Practical Implications

This research has several practical implications. First, parents of only children should consider their child’s need for peer interactions to develop their cooperative orientation, which will benefit their child’s social adaptation and career success (e.g., being a leader in group settings). To reduce the negative effects due to the lack of sibling interactions, parents may be encouraged to create other teamwork opportunities for their children, such as group activities at school and summer/winter camps.

Secondly, parents should consider the detrimental effect of their child’s parental dependency on their growth and should be encouraged to find a healthy balance between laissez-faire and over interference. Parents should be encouraged to treat their children as friends, try to understand them, avoid dominating communication and decision-making during child-parental interactions, and give them opportunities to solve problems by themselves.

Finally, schools and companies could provide education and training programs for students or employees to improve their social skills. For example, schools may offer courses on how to establish rapport with others, cooperate with others, work in teams, and act as a team leader. Moreover, companies could build a teamwork culture and provide training to develop employees’ teamwork and leadership skills. Such practices will not only benefit individual development but also enhance organizational effectiveness.

### Limitations and Future Directions

This study has several limitations. First, the two samples were from China, and the high collectivism in Chinese people (Hofstede, 2001) may contribute to high cooperative orientation and low competitive orientation (Leibbrandt et al., 2013; Kadoya et al., 2018). Indeed, our results showed that the self-reported competitive orientation is lower than cooperative orientation, which implicitly supports the social desirability effect mentioned by Chen et al. (2011). However, our findings showed that even in a context that values collectivism and teamwork, the cooperative orientation of only children was lower than that of non-only children. These findings demonstrated that, cooperative orientation makes a difference between only and non-only children in power acquisition. To further explore the role of cooperative and competitive orientation, we recommend future research to test the hypotheses in a different context, such as in an individualistic culture.

Secondly, our study demonstrated the disadvantages that only children have in terms of power acquisition from the perspective of cooperative orientation. An underlying assumption is that individuals are afforded power for their cooperative behaviors and contributions to the team (Keltner et al., 2008). However, individuals may acquire power within group settings for other reasons, such as organizational politics, impression management, and charisma. Thus, exploration of other perspectives to explain the difference between only and non-only children is needed in future studies.

Thirdly, the main effect of only child status on power acquisition is supported in Study 1 but not in Study 2. This suggested the existence of moderating factors that influence the effect of only child status on power acquisition. The interdependent relationship between individuals and other group members may be one of the factors that contribute to this difference. As employees in organizations, participants in Study 1 are usually more interdependent with their coworkers than are students with their schoolmates or classmates in university (Study 2). Interpersonal skills and influence tactics maybe more important in the highly interdependent context than in the lower interdependent context for individuals to emerge as leaders or acquire power. Therefore, the disadvantage of only children is more salient in the workplace. We recommend future studies to explore this possibility.

### Research Conclusion

We revealed that (1) compared with non-only children, only children were disadvantaged in terms of power acquisition; (2) cooperative orientation plays a mediating role between only child status and power acquisition; (3) the difference in cooperative orientation between only and non-only children was smaller when dependency on parents was high, whereas it became larger when dependency on parents was low. These findings have several theoretical and practical implications for research on power acquisition and only children.

## Data Availability Statement

The raw data supporting the conclusions of this article will be made available by the authors, without undue reservation.

## Ethics Statement

The studies involving human participants were reviewed and approved by the Research Committee of Shanghai University of Finance and Economics. The patients/participants provided their written informed consent to participate in this study.

## Author Contributions

All authors listed made a substantial, direct, and intellectual contribution to the work, and approved it for publication.

## Conflict of Interest

HB was employed by Shanghai Bihe Biochemical Technology Co., Ltd. The remaining authors declare that the research was conducted in the absence of any commercial or financial relationships that could be construed as a potential conflict of interest.

## Publisher’s Note

All claims expressed in this article are solely those of the authors and do not necessarily represent those of their affiliated organizations, or those of the publisher, the editors and the reviewers. Any product that may be evaluated in this article, or claim that may be made by its manufacturer, is not guaranteed or endorsed by the publisher.

## Footnotes

1 This study was based on a larger dataset. To reduce the cognitive load of the participants, we divided the data collection process into several parts in which we measured the independent variables, mediators, moderators, and dependent variables at different time points. Thus, we were able to also reduce common method bias. The same procedure was applied in Study 2.

2 Hukou is a system of household registration used in mainland China. The registry contains each individual’s basic status information, such as urban or rural residency. In China, urban residents generally have a higher economic status than do rural residents; therefore, their children receive a better education. This is also an important factor that influences power acquisition. Thus, we considered hukou as a control variable.

3 Although we did not directly measure whether participants value cooperative behaviors over competitive behaviors, our data provided indirect evidence that it is socially desirable for Chinese people to be cooperative rather than competitive. For example, in Study 1, self-reported cooperative orientation (*M* = 4.01, *SD* = 0.55) was higher than competitive orientation (*M* = 3.38, *SD* = 0.65), *t*[154] = 8.78, *p* < 0.001). Similarly, in Study 2, cooperative orientation (*M* = 3.47, *SD* = 0.60) was higher than competitive orientation (*M* = 3.03, *SD* = 0.56), *t*[374] = 11.83, *p* < 0.001). This implicitly supports the social desirability effect (Chen et al., 2011).
